# The demand for intermittent preventive treatment of malaria in pregnancy using sulfadoxine-pyrimethamine in the Volta Region of Ghana

**DOI:** 10.1371/journal.pone.0308321

**Published:** 2024-09-05

**Authors:** Livingstone Asem, Abdul-Gafaru Abdulia, Patrick Opoku Assuming, Gordon Abeka-Nkrumah

**Affiliations:** 1 Department of Public Administration and Health Services Management, Business School, University of Ghana, Legon-Accra, Ghana; 2 Deparment of Health Policy Planning and Management, Fred N. Binka School of Public Health, University of Health and Allied Sciences, Ho, Ghana; King Caesar University, UGANDA

## Abstract

**Background:**

Malaria in pregnancy (MiP) is a preventable condition leading to maternal and neonatal morbidity and mortality. Invariably, with all the knowledge about the serious consequences of MiP for the woman, the unborn child, and the neonate, the uptake of Intermittent Preventive Treatment of Malaria in pregnancy using sulfadoxine-pyrimethamine (IPTp-SP) is low in most malaria-endemic countries, including Ghana. This study sought to examine the uptake and service user predictors of the implementation of IPTp-SP after the policy upgrade in 2014.

**Methods:**

This cross-sectional survey was carried out in two selected districts in the Volta Region. The study participants were randomly selected from communities within Nkwanta North and North Tongu District. A total of 438 mothers who have delivered in the past 24 months were selected for the study. The women were interviewed on their background, knowledge, and attitude toward the use of IPTp-SP using a structured questionnaire. Multiple logistic regression was done to determine the factors that influence the demand for IPTp-SP. The results were presented in the form of tables.

**Results:**

The mean number of antenatal care (ANC) attendance was 5 (SD:2.6) visits per client, with 262 (59.82%) of them getting the 3+ doses of IPTp-SP. Also, a significant 44 (10.1%) of the mothers did not receive any dose of IPTp-SP. Respondents who attended antenatal clinics 4–7 times had 7 (CI:3.9–12.3) times higher uptake of 3+ doses of IPTp-SP as compared to others who attended less than 4 visits. Similarly, women who had 8 or more visits had a 16.1 (CI: 5.9–43.6) times higher chance of getting more than 2 doses of IPTp-SP compared with others who had fewer than 4 attendances.

**Conclusion:**

The uptake of 3+ doses of IPTp-SP is still lower than the global target of 80%. Thus, the need for innovative interventions aimed at improving antenatal attendance and early booking for IPTp-SP are recommended.

## Background

Infection with malaria during pregnancy is of significant public health interest in malaria-prevalent regions with steady transmissions, such as tropical Africa. Infections from malaria in the course of pregnancy can cause many unfortunate health effects on pregnant women and their newborns, such as maternal anemia, underweight babies, and preterm delivery [1–3]. Low birth weight is the highest risk predictor for newborn mortality and a key contributor to the mortality of children under one year [4–6].

Malaria infection during pregnancy can be prevented using policies such as intermittent preventive therapy (IPTp), proper case management, and the sharing of insecticide-treated bed nets in endemic regions all over the world. Despite that, just a segment of these pregnant women receive these useful malaria medicines [7].

WHO in 2010 developed guidelines that endorsed microscopy diagnosis or rapid diagnostic test (RDT) for all individuals infected by suspected malaria and this includes pregnant women. Since pregnant women experience other health conditions similar to MiP, proper management of MiP through diagnostics confirmation is very crucial. WHO in a recent policy upgrade, further recommended effective case management and prevention by using long lasting-insecticide bed-nets (LLiNs), and intermittent preventive treatment (IPTp) with sulfadoxine-pyrimethamine for the prevention of malaria disease during pregnancy, delivered through antenatal care (ANC) platform [8].

Malaria in pregnancy is projected to be the cause of up to 200,000 infant deaths in Africa yearly. It also causes both preterm births and intrauterine growth retardation. In some cases surviving infants frequently undergo lasting consequences from infection in the womb that hinder their growth and advancement [9, 10]. It was estimated in 2010 that 11.4 million pregnancies in Sub-Saharan Africa (41% of the estimated 27.6 million live births) could have contracted P. falciparum placental disease at some phases of pregnancy with the absence of malaria prevention intervention during pregnancy [11, 12]. Also, from the global malaria report 2019, it was stated that among 36 African countries that reported on IPTp-SP in 2018, only 31% of qualified pregnant women were given the proposed 3 or more doses of IPTp-SP as compared with 22% in 2017 [13].

Inside Ghana, malaria illness during pregnancy constitutes a significant public health challenge. For example, malaria among pregnant women in Ghana represents about 16.8% of hospital admissions and 3.4% of total deaths. Thus, malaria infections among pregnant women constitute a tremendous burden on the country’s health system [14].

Ghana, implementing the proposal of the World Health Organisation in 2000, approved fresh malaria management guidelines in 2004 which were reviewed in 2007, and again, in 2012. Accordingly, Ghana changed from the usage of monotherapy to combination treatment using artemisinin-based combination therapy (ACT) and portions of these guidelines were the shift from the practice of weekly Chloroquine chemoprophylaxis to Sulphadoxine–Pyrimethamine as an intermittent treatment for malaria infections during pregnancy [15, 16]. Intermittent preventive therapy during pregnancy (IPTp), is grounded in the belief that each pregnant woman in a malaria-endemic zone has malaria parasites in her placenta, whether or not she has symptoms of malaria. Sulphadoxine-pyrimethamine is a single-dose antimalarial medicine that has been realized to be very beneficial in stopping malaria during pregnancy and decreasing the magnitude of malaria disease in mothers and babies [17]. In 2014, Ghana reviewed its strategy on IPTp‐SP to mirror this revised strategy of the WHO in 2012 [14, 18]. Thus, Ghana has since 2014 implemented the updated policy on IPTp-SP.

Although there are several studies within the general malaria prevention literature that have examined the clinical efficacy of IPTp-SP like van Eijk et al. and Megnekou et al. [19, 20]. In addressing malaria in pregnancy, there is nonetheless limited information on the demand for IPTp-SP in Ghana. Most of the earlier studies examined the demand-side predictors reported that late timing of antenatal attendance, low awareness of IPTp-SP, and location to health facilities were some of the issues inhibiting the uptake of IPTp-SP [21–24]. This research, therefore, seeks to examine the predictors of uptake using maternal and child health care in the Volta Region as a case study to assess the demand for IPTp-SP policy in Ghana.

## Methods and materials

### Study design and setting

A quantitative, cross-sectional study was conducted among women with children less than two years in the Volta Region (Volta and Oti) of Ghana. The Volta Region was one of Ghana’s ten (10) administrative regions at the beginning of the study which was later split into sixteen (16) administrative regions. It is situated in the eastern part of the country sharing boundaries in the north with the Northern region, the south with the Gulf of Guinea, in the west with the Volta Lake, and in the east with the Republic of Togo. From the routine service, the region had IPTp3 Coverage of 50.6% in 2019, with Nkwanta South having the lowest coverage of 29% and North Tongu District having the highest coverage of 76.1%. However, comparing the average coverage from 2015 to 2019, Nkwanta North had the lowest coverage of 23.1% with North Tongu having the highest coverage of 59.5%, and both districts were selected for the study. The Volta Region was selected because it presents a peculiar and widespread case of the problem under investigation. Based on data from the Policy, Planning, Monitoring and Evaluation Directorate (PPMED) of the Ghana Health Service (GHS), the Volta Region was ranked 10^th^ (last) on the “Performance Gauge” (PG) scale in 2016 and 2017. The Holistic Assessment of the Health Sector Programme of Work 2014 and the DHS report of 2014 mentioned the Volta Region as an area that needs special attention. The region is, therefore, seen as a classical area for determining the demand-side predictors of intermittent preventive treatment of malaria in Ghana [25].

### Study population

**T**he study population includes all women who gave birth twenty-four months preceding the survey from the communities within the region expected to have used antenatal services. The survey date was 1^st^ June 2020 to 10^th^ July 2020.

### Inclusion and exclusion criteria

#### Inclusion

All women who gave birth twenty-four months preceding the survey from the communities within the region in the two selected districts and are available at the time of the study.

#### Exclusion

This excludes all women who gave birth long before the past 24 months in the two selected districts and those who were not available at the time of the study.

### Variables

Based on the information reviewed from earlier studies done elsewhere. This study included the following variables that have been linked in theory and empirically to malaria in pregnancy and the use of IPTp-SP. The outcome/dependent variable was the uptake of 3+ doses of IPTp-SP by women exposed to the policy and independent/explanatory variables included both individual-level demographic characteristics of the respondents and IPTp-SP implementation-related characteristics. This includes age, marital status, educational level of woman, occupation of the woman, religion, ethnic group, partner education, partner occupation, partner age, duration of the marriage, monthly household income, household assert, means of transportation to the nearest health facility, estimated traveled time to the nearest health facility, estimated distance to the nearest health facility, health insurance enrollment, and health insurance validity status, gestational age booked for antenatal, gestational age for IPTp-SP, gravida, parity, number of live births, and number of antenatal visits.

### Sample size determination and sampling

The formula for sample size determination based on the Yamane (1967) approach is stated as follows [26]

$$n=\frac{N}{1+N({e)}^{2}}$$ Eq 1

Where n is the sample size, N is the population size, e is the level of precision. Yamane’s (1967) approach to sample size determination assumes that the population of the study is known and is finite. The sample frame (target) population is estimated to be 76,341 for the entire Volta Region. The appropriate sample size is determined based on Yamane’s (1967) approach at the precision of 5% as presented below [26]:

$$n=\frac{76,341}{1+76,341({0.05)}^{2}}=398$$ Eq 2

The required sample size that was selected is therefore 398. Given the possibility that not all the target respondents were reached (non-response), 10% of the obtained sample size, four hundred and thirty-eight (438), were added to compensate for persons that the researcher was unable to reach.

The sampling procedure was adapted from the 2014 Ghana Demographic and Health Survey (GDHS) and followed a two-stage sample design intended to allow estimates of key indicators for urban, and rural areas in the two selected districts. The first stage using probability proportional to the size and then thirty households per cluster scenario [27], the researcher selected eight (8) clusters for North Tongu and six (6) clusters for Nkwanta North, giving a total of fourteen(14) clusters for the study area with five (5) urban and nine (9) rural. The second stage of sampling involved the selection of the sampling unit for enumeration after listing all members of each household in the enumeration area. All the eligible women were sampled based on the population proportion to the size of the eligible population contribution after the listing of all the members of the households in the clusters and then a random selection of the eligible population was done using Stata to select the respondents. In all, 438 women were selected at random for the interview. However, those that were not available at the time of the enumeration were replaced by the next in the randomized order.

### Data collection and quality management

The study used a structured questionnaire for data collection. The questionnaire solicited data on the demographics and use of IPTp-SP. Data on the number of antenatal visits, number of doses of IPTp-SP, parity, and gravida was obtained from the questionnaire and triangulated by their maternal health records. Data was captured using Kobo collect app, data extracted as a Microsoft Excel file, and exported to STATA 14.1 for analysis. Data cleaning was done at every stage to ensure that we had good-quality data for analysis.

### Data analysis

In the analysis, the continuous independent variables were categorized into intervals range as required whilst the categorical variables such as sex, educational level, ethnicity, etc. retained their categorization during analysis. To ascertain the wealth index, the researcher had to first conduct a principal component analysis of the household assets and possessions to establish which variable should be included in the model to determine the wealth of the household. In the study, data was collected on household assets and possessions which may be correlated in an unknown and complex way. Thus, when a set of variables are correlated in a complex and unknown way along several dimensions, Principal Component Analysis (PCA) is employed to reduce these variables by assessing which variables behave similarly. Based on the variables and their relationship to each other, PCA creates a new set of variables called the principal component. The household assets and possessions collected during the study were assigned weights based on the PCA and the resulting scores were standardized to a standard normal distribution. Based on these aggregate scores, individuals and their household wealth were put into five quintiles.

The chi-square test of associations was used to check for dependence between the sociodemographic variables and the outcome variables. All independent variables with p<0.05 in the bivariate analysis were included in the multivariable logistic regression to further examine the association between the outcome and each independent variable while controlling the effect of other explanatory variables by calculating adjusted odds ratios (AOR). The level of significance used was 5% (0.05), two-tailed at 95% confidence interval (CI).

The goodness of fit of the model was tested by using the criterion-based method, usually known as the “Leaps-and-Bounds algorithm”, which was used in the variable selection because it involved a wider search and compared models in a preferable manner. It also identified the subset of variables with the most discriminatory power (predictive sense) is proposed. It minimized the parametric estimator of the error rate among all possible variable subsets, evaluating only a fraction of the total number of subsets. These criteria took into consideration the nature of the problem and the objective of the analysis.

To investigate the effect of the predictor variable on the outcome variables, binary logistic regression analysis was done at two levels. The first level was to establish the crude level of association between the independent variable and the dependent variables for the study. For this study, all independent variables were added to the model to establish the crude relationship with the dependent variables at various levels of measurement in accordance to the study objectives. Afterward, variables that were significant in the first model at the bivariate analysis level were moved to another model to adjust for the effects of those variables. A p-value < 0.05 was considered statistically significant.

### Ethical approval and consent to participate

Ethical clearance was obtained from Ghana Health Service’s Ethical Review Committee (ERC) with protocol ID No: GHS-ERC 005/12/19 through the University of Ghana, Business School. The researcher obtained written informed consent and assent were obtained from each study participant whose age was 18 years and above and below 18 years respectively. Willingness to participate in the study and parental permission was confirmed by signing or fingerprinting on the informed consent form.

## Results

### Sociodemographic characteristics of the service users

The average age of the respondents was 29 years (SD:6.1) with 211 (54.8%) of them falling within the 20–29 age group, 237(54.1%) of the respondents, had lower than Junior High School/Junior Secondary School/Middle education. Christians, 353 (80.6%), formed the majority of the religious groupings. Seventy-eight percent (77.6%), were married. With 155(35.39%) of the respondents were Farmers. The most dominant ethnic group was Ewes, who constituted 54.79% of the respondents. Most of the partners of the women, 196 (44.7%), were between the ages of 30 and 39 with a mean age of 35 years (SD:7.6). One hundred and eighty-nine (43.2%) of the partners of the respondents had less than JHS/JSS/Middle educational and 187(42.69%) of them were farmers. Most of the respondents, 242 (55.25%), had been married for 3 to 9 years with an average duration of 7 years (SD:4.9). An average household income for the respondents was GH ¢349($34.9) per month with a majority of them, 243 (55.48%), earning between 100($10) to 999($99) Ghana cedis per month. The highest wealth index quintile was the second with 90 (20.55%) of the women in this category. This was computed for the list of assets like owning a mobile phone, a television set, radio, bed, farmland, means of transport, a house, etc. The majority of the respondents, 245 (55.9%), resided in the rural areas of the region with only 193 (44.1%) in the urban areas. 210 (47.95%) of the respondents, walked to the nearest health facility during antenatal care with the average estimated travel time and distance being 32 minutes (SD:21.0) and 8km (SD:9.1) respectively. A high number of the respondents, 282 (64.68%), booked for antenatal care in the first trimester. An overwhelming majority of them, 408 (93.15%), had enrolled in the National Health Insurance Scheme. However, only 221 (50.46%) of them were active to access free services. The majority of the women, 237 (54.1%), had a parity of 0 to 2 pregnancies, the average gravida was 3 (SD:1.9) pregnancies and the average number of live births was 3 (SD:1.8) children as shown in Table 1 below.

**Table 1:** *Background characteristics of IPTp-SP service users*.

Item	Number N = 438	Percentage (%)
**Age group(women)**		
Below 20	20	4.6
20–29	211	48.2
30+	207	47.2
**Educational level (woman)**	** **	** **
<Middle/JHS/JSS	237	54.1
Middle/JSS/JHS	134	30.6
>Middle/JHS/JSS	67	15.3
**Religious affiliation (woman)**		
Christians	353	80.6
Muslim	23	5.3
Others	62	14.2
**Marital Status (woman)**		
Co-habitation	60	13.7
Married	340	77.6
Others	38	8.7
**Occupation (woman)**		
Farmer/Agriculture	155	35.4
Government worker	19	19
Trader/Artisans	223	36.2
Unemployed	41	9.4
**Ethnic Group (woman)**		
Ewe	240	54.79
Guan	9	2.05
Konkomba	137	31.28
Other	52	11.87
**District**		
Nkwanta north	182	41.55
North Tongu	256	58.45
**Partner Age**		
20–29	104	43.2
30–39	196	44.7
40+	138	31.5
**Educational level (Partner)**		
<Middle/JHS/JSS	189	43.2
Middle/JSS/JHS	123	28.1
>Middle/JHS/JSS	126	28.8
**Occupation (Partner)**		
Farmer/Agriculture	187	42.69
Government workers	56	12.79
Trader/Artisans	190	6.16
Others	5	19.86
**Duration of marriage**		
<3	82	18.72
3–9	242	55.25
10+	114	26.03
**Monthly Household Income**		
<100	156	35.62
100–999	243	55.48
1000+	39	39
**Wealth Index**		
Lowest	88	20.09
Second	90	20.55
Middle	85	19.41
Fourth	88	20.09
Highest	87	19.86
**Residence**		
Urban	193	44.06
Rural	245	55.94
**Means of Transport**		
Boat	1	0.23
Motor bike	164	37.44
Vehicle	63	14.38
Walking	210	47.95
**Estimated travel time to the nearest health facility(One-Way)**		
<30mins	276	60.96
31-60mins	156	35.62
1hr+	15	3.42
**Estimated distance to the nearest health facility**		
<3km	143	32.72
3-5km	105	24.03
6+km	189	43.25
**Gestational Age at Booking for ANC**		
1st Trimester	282	64.68
2nd Trimester	131	30.05
3rd Trimester	23	5.28
**NHIS enrollment Status**		
Not enrolled	30	6.85
Enrolled	408	93.15
**Validity of NHIS card**		
Not active/never enrolled	217	49.54
Active	221	50.46
**Number of pregnancies(gravida)**		
<3	208	47.49
3–4	137	31.28
5+	93	21.23
**Parity**		
0–2	237	54.1
3–4	124	28.3
5+	77	17.6
**Livebirths**		
0–2	236	53.9
3–4	126	28.77
5+	76	17.35
**Number of ANC Visits**		
<4	100	22.83
4–7	264	60.27
8+	74	16.89
**Doses of SP Received**		
No dose	44	10.05
1–2 doses	123	30.14
3+ doses	262	59.82
**Gestational age at booking for IPTp-SP**		
<17 wks	221	50.46
17-24wks	145	33.11
25wks	72	16.44

### Uptake of 3 plus doses of IPTp-SP

The mean number of antenatal care (ANC) attendance was 5 (SD:2.6) visits per client, with 262 (59.82%) of them getting the 3 plus doses of IPTp-SP while 44 (10.1%) of the respondent did not receive any dose of IPTp-SP. Also, 221 (50.46%) were booked for IPTp-SP before the 17^th^ week of pregnancy as shown in the Table 1 below.

### Crosstabulation of background variables and uptake of 3+ doses IPTp-SP

For the IPTp-SP uptake for prevention of MiP against sociodemographic variables, only one variable out of 25 was significant at (p<0.05) and thus had an association with the uptake of 3 plus doses of IPTp-SP. Some variables like age of women, marital status, ethnic group, partner’s age, district of residence, gestational age at booking for ANC, National Health Insurance Scheme (NHIS) card availability, NHIS card active, gravidity, parity, number of livebirths, number of antenatal visits and gestational age at booking for IPTp-SP, which are known in the literature to influence uptake, had no association with the uptake. Women who attend antenatal in the first trimester are more likely to get 3+ doses of IPTp-SP as shown in the Table 2 below:

**Table 2:** *Association between sociodemographic variables and uptake of 3+ doses of IPTp-SP*.

Variables	Number N = 438	<3 doses	3+ doses	Ch12	p-value
**Age group**				4.9	0.522
Below 20	20	4.2	2.8		
20–29	211	55.1	46		
30+	207	40.7	51.2		
**Educational level (woman)**				7.5	0.162
< JHS/JSS	237	51.5	61.3		
JHS/JSS	134	34.4	22.7		
SSS/SHS +	67	14.1	16		
**Religious affiliation (woman)**				0.5	0.081
Christians	353	87	86.7		
Muslims	23	3.6	2.6		
Others	62	9.5	10.7		
**Marital Status (woman)**				1.9	0.609
Co-habitation	60	12.6	12.2		
Married	340	7.7	4.6		
Others	38	79.7	8.3		
**Occupation (woman)**				19.17	0.158
Farmer	155	49.7	52.4		
Government workers	19	2.1	7.8		
Trader/Artisan	223	35.1	36.2		
Unemployed	41				
**Ethnic Group (woman)**				13.7	0.297
Ewe	240	45.9	36.4		
Guan	9	0.8	6.7		
Konkomba	137	40.3	46.9		
Other	52	13	10		
**Partner Age**				1.1	0.888
20–29	104	23	21.7		
30–39	196	43.3	48.2		
40+	138	33.7	30.1		
**Educational level (Partner)**				10.8	0.085
<JHS/JSS	189	45.8	54.8		
JHS/JSS	123	32.5	18.8		
>JHS/JSS	126	21.7	26.4		
**Occupation (Partner)**				5.9	0.303
Farmer/Agriculture	187	43.8	55.3		
Government worker	56	15.7	11.3		
Trader/Artisan	190	40.2	33.2		
Others	5	0.3	3		
**Duration of marriage**				16.9	0.116
<3	82	18.9	11.7		
3–9	242	50.4	69.6		
10+	114	30.7	18.7		
**Monthly Household Income**				5.5	0.36
<100	156	38.8	47.3		
100–999	243	58.1	47.3		
1000+	39	3.1	5.4		
**Wealth Index**				18.1	0.28
Lowest	88	20.3	14.3		
second	90	17.3	20.6		
middle	85	20.8	23.2		
fourth	88	34.4	23.5		
highest	87	7.2	18.4		
**Residence**				1.9	0.202
Urban	193	82.9	77.6		
Rural	245	17	22.4		
**District**				3.1	0.352
Nkwanta North	182	52.1	60.5		
North Tongu	256	47.9	39.6		
**Means of Transport**				13.7	0.114
Boat	1	0.2	0		
Motor bike	164	40.6	25.2		
Vehicle	63	5.4	10.2		
Walking	210	53.9	64.7		
**Estimate travel time to nearest health facility**				1.8	0.554
<30mins	276	64.6	70.6		
>30mins	156	35.3	29.4		
**Estimated distance to nearest health facility**				16.4	0.26
<5km	184	25.3	41.1		
5-9km	162	59.8	40.9		
10+km	92	14.9	17.9		
**Gestational Age at Booking for ANC**				26.02	0.065
1st Trimester	282	53.5	75.2		
2nd Trimester	131	39.9	23.6		
3rd Trimester	23	6.6	1.1		
**NHIS card availability**				0.97	0.430
no card	30	9.2	6.7		
card available	408	90.8	93.3		
**Validity of NHIS card**				7.1	0.305
not active	217	53.6	40.8		
active	221	46.4	59.2		
**Gravidity**				5.9	0.412
<3	208	40.8	47.5		
3–4	137	28.9	32.3		
5+	93	30.3	20.2		
**Parity**				5.01	0.557
0–2	237	50.5	56.9		
3–4	124	22.9	24.5		
5+	77	26.6	18.9		
**Number of Livebirths**				5.34	0.517
1–2	236	50.6	56.7		
3–4	126	22.8	25.2		
5+	76	26.7	18.3		
**Number of ANC Visits**				69.1	0.000
<4	100	41.7	8.3		
4–7	264	53.9	79.9		
8+	74	4.4	11.7		
**Gestational age at booking for IPTp-SP**				10.5	0.189
<17 wks	221	56.4	71.3		
17-24wks	145	28.4	18.1		
25wks	72	15.1	10.6		

N: represents frequency, p<0.05

### Logistic regression for uptake of optimal doses of IPTp-SP

From the crosstabulation (Table 2) above, only the number of antenatal attendances had a connection with the IPTp-SP uptake (p<0.05). Thus to select the model with goodness of fit, the criterion-based method, usually known as the “Leaps-and-Bounds algorithm”, was used. Education level of the women was included based on information from the literature indicating that higher women’s education is associated with uptake of IPTp-SP. Thus, controlling for confounding variables, the optimal model selected among the 4 nested models was model 2 (AIC = 532.2588). The predictors selected are the number of antenatal care visits and maternal education. The number of antenatal attendances was connected with the uptake of 3 plus doses (optimal doses) of IPTp-SP. Overall, the respondents who attended the antenatal clinic 4–7 times had 7 (CI:3.9–12.3) times higher uptake of 3+ doses of IPTp-SP as compared to others who attended less than 4 visits. Similarly, women who had 8 or more visits had a 16.1 (CI: 5.9–43.6) times higher chance of getting more than 2 doses of IPTp-SP as compared with others who had fewer than 4 attendances as shown in Table 3 below:

**Table 3:** *Crude and adjusted associations between variables and uptake of 3+ doses of IPTp-SP*.

Variables	Crude Odd Ratio (COR)	95%CI	p-value	Adjusted Odd Ratio(AOR)	95%CI	p-value
**Number of ANC Visits**						
<4	1			1		
4–7	7.4	4.2–13.2	0.000	7.0	3.9–12.3	0.000
8+	13.4	6.7–26.7	0.000	16.1	5.9–43.7	0.000
**Educational level (woman)**						
<Middle/JHS/JSS	1			1		
Middle/JSS/JHS	0.6	0.3–1.2	0.106	0.5	0.2–1.4	0.195
>Middle/JSS/JHS	1	0.5–1.9	0.887	0.8	0.3–2.2	0.62

**Crude Odds Ratio(COR):** odds ratio of one independent variable predicting the dependent variable

**Adjusted Odds Ratio(AOR):** holds other relevant variables constant and provides the odds ratio from the potential variable of interest which is adjusted for the other independent variables included in the model. CI: confidence interval, p<0.05.

## Discussion

### Discussion of the predictors of IPTp-SP uptake

One of the major objectives of this study is to examine the magnitude and the reasons related to the uptake of 3+ doses of IPTp-SP by women in the Volta Region after the policy update by WHO in 2012. The research has revealed that generally, the percentage of women who took 3+ doses of IPTp-SP was 59.8%, which is nevertheless extremely lower in the surveyed populace as compared to the Roll Back Malaria (RBM) aim of a minimum of 80% for all pregnant women living in regions with high malaria spread [28]. Among the twenty-five (25) covariants that were examined to describe the of 3+ doses of IPTp-SP uptake among pregnant women in the Volta Region in Ghana, only the number of antenatal attendance was discovered to influence the uptake of IPTp-SP after modifying other determinants. The explanatory examination of this enquire reveals that the percentage of the women that receive 3+ doses of IPTp-SP is the highest within women making 4 or more antenatal care attendance than those making less attendance. Likewise, the corrected odds ratio scores indicate that pregnant women who visit antenatal care less than 3 times during gestational time had a lesser chance of taking maximum doses of IPTp-SP likened to pregnant women who had completed 4+ attendance. Thus, with the new requirements of making 8 visits or more, pregnant women who attend ANC 8 times or more have higher odds of getting the maximum 3+ dose as likened to pregnant women who had just 4 attendances. For pregnant women who booked for IPTp-SP later after the 16^th^ week of gestation, the adjusted odds ratio shows that these women have lesser odds of getting the maximum doses of IPTp-SP as compared to individuals who book for antenatal care earlier. This result is coherent with research in Malawi [29, 30], Uganda [21], Tanzania [23, 31], Ghana [32] and Burkina Faso [33]. Antenatal care clinics offer a stage for vital health packages and services such as health promotion, prevention, testing, and identification of illness, all intended at enhancing the health of pregnant women and their unborn children must be intensified [34]. Using IPTp-SP is one of the prevention strategies being executed at the antenatal care in the Volta Region. The 2012 update policy recommended 3+ doses of IPTp-SP and the first dose should be received as timely as feasible through the second trimester of gestation [35]. This suggests that timely ANC commencement would boost the likelihood of getting the maximum IPTp-SP doses as there is a probability of more antenatal clinic attendance to be made. In this study, 64.8% of the women commence antenatal clinic attendance during their first trimester, which agrees with the WHO recommendations that pregnant women must begin antenatal clinic attendance during the first trimester of pregnancy [36]. The results from the research also reported that less than half of the study respondents visit the antenatal clinics at least 8 times, implying the slow-paced of use of focused antenatal care (FANC) strategy was utilized at the period of the research as a guide. FANC strategy proposed at least eight (8) antenatal care attendance and examination by a skilled health provider before delivery [37]. The reviewed WHO policy was designed to match with the FANC Model in order to boost the maximum doses of IPTp-SP uptake. However, only 11.7% of pregnant women who made eight-plus attendance get the maximum doses of IPTp-SP. This is an overlooked chance to give the proposed maximum doses of IPTp-SP to women who attend antenatal at least 8 times before delivery [38].

The difference concerning the proportion of 8+ antenatal visits and the 3+ doses of IPTp-SP uptake could occur partially because of recurrent unavailability of SP in the antenatal care clinics. Additional clarifications might be that certain antenatal clinic attendance was not planned because of unforeseen problems; thus IPTp-SP doses were not given.

### Limitations

Geographically, the scope of the study covers only the Volta Region (Volta and Oti) of Ghana, specifically the two purposively selected districts (North Tongu and Nkwanta North). In terms of the study unit, the scope was also narrowed to women who had given birth in the past 24 months. The focus of the study was on IPTp-SP policy and not on any other malaria intervention. Another possible limitation came from the use of questionnaires. The reliability of the data from the questionnaire depended on the skills and experience of field enumerators Research Assistants (RAs) that conducted the interviews as well as the extent to which the respondents could remember facts and events. Also, only well-trained graduates were used in the data collection to reduce errors. Furthermore, the study had financial challenges, hence the choice of only two districts for the study in the Volta Region. What this means is that the findings of the study should be interpreted with caution because the two participating districts were purposively selected based on low and high coverage of IPTp-SP [39].

## Conclusion

Malaria in pregnancy continues to be a major health challenge in Ghana after the adoption the malaria treatment policy which includes IPTp in 2004. The IPTp-SP policy was upgraded to mirror the present policy of the WHO in 2012, however, Ghana has never achieved the global target of 80% for optimum doses of IPTp-SP. Over the past years, this issue on the uptake of IPTp-SP is gaining popularity in public health research. The study demonstrated that there is low IPTp-SP uptake in the study population and this seems to be associated with the number of antenatal attendances. Therefore, community-based health promotion on timely and regular antenatal attendance to promote the benefit of IPTp-SP should be encouraged. Further research needs to be carried out to understand the provider side predictors influencing the uptake of 3+ doses of IPTp-SP.
