# Supplementary material for: The demand for intermittent preventive treatment of malaria in pregnancy using sulfadoxine-pyrimethamine in the Volta Region of Ghana
Source: PLoS One. 2024 Sep 5;19(9):e0308321. doi: 10.1371/journal.pone.0308321 (PMC11376531; doi:10.1371/journal.pone.0308321)
Supplement: Attachment — Submitted filename: Review on the study conducted in Ghana _IPTp-SP.docx [file pone.0308321.s001.docx]

I appreciate the opportunity to review the manuscript with the title “*The demand for intermittent preventive treatment of 5 malaria in pregnancy using sulfadoxine-pyrimethamine in 6 the Volta Region of Ghana.*” I commend the authors for a number of strengths of their work, including:

1. Managing to conduct this study given that, they did not have specific funding support to implement it.
2. The study aims of assessing predictors of IPTp-SP uptake which is one of the major challenges facing most malaria endemic countries towards meeting the set nationals and global targets. Observed issues will help informing the policy towards reviewing the existing strategies
3. Managed to articulate key predictors to determine their relationship with outcome variable

However, given the strengths which I have indicated, as I read the manuscript, I noted some areas in which I would have appreciated more clarification. I hope the paper could be further improved on:

1. Abstract:

Line number 53 can be rephrased to “*This cross-sectional survey was carried out in two selected districts in the Volta Region.”*

Line number *58:* What has been done in your work is multiple logistic regression because it involves single outcome and several explanatory variables. Therefore, I recommend to mention “*Multiple* logistic regression analysis was used to determine …

2. Background section:

After going through the background section, I realized that a lot of information have been provided of which some are too general and not specific for the manuscript aim.

Line 75 - 93, provide details for malaria in general, which can distract a reader who is interested in malaria in pregnancy. Therefore, I would recommend to focus on the scope of the paper

Line 117 – 118, the provided figure does not have reference to support it. Maybe it was an oversight.

Line 121 – 122, key references are missing

Line 126 – 127, authors indicated that IPTp-SP was introduced because it is “*grounded in the belief that each pregnant woman in a malaria-endemic zone has malaria organisms in her blood and/or placenta.”* This does not seem to be the case. A lot of evidences have been generated indicating malaria parasites tending to harbor in the placenta. So, providing SP helps to reduce unwanted outcomes among others severe maternal anemia, miscarriages, underweight etc.

3. Methods section:

Line 154 – 158, and 162 – 166: These lines does not seem to be part of the methodology section; rather I would recommend to move them to the background. This is why background was overcrowded with lots of details which are not specific to the study topic while missing this crucial information.

Line 192: I understand the provided formula was used to determine studied informants. However, I was wondering how this formula factored in the issue of the current status of IPTp uptake in the study area.

Where is the source of the indicated level of precision?

Line 195: The indicated sample frame (target) population which you indicated was estimated to be 76,341; kindly specify whether this is for entire Volta region or just in the studied districts.

In this section, I could not find how the surveyed districts were selected though, I noted in the conclusions that the districts were purposively selected

4. Results section:

Line 275: JHS/JSS this abbreviation appeared for the first time and I could not find anywhere written in its long form

Line 310: You indicated maternal age to have no effect on the uptake. However, in Line 314 – 316; you mentioned that “the higher the age the more the uptake. So, please could you please cross-check this to avoid confusing the reader.

Line 340, 341: write in complete form in the bracket e.g., instead of *Crude odds ratio (OR)*; write Crude odds ratio (COR) and for adjusted as well

5. Discussion section:

Line 364 – 367: This section you indicated pregnant women booking with gestational age above 16 weeks; their odds of getting 3+ doses were low. Again, this is contrary to what you indicated in Line 311 which you mentioned gestational age at booking for ANC to have no effect on the uptake. Kindly reconfirm your bivariate outputs.

6. Limitation section:

Line 400: Ras in mentioned for the first time with no long form
