# Supplementary material for: The demand for intermittent preventive treatment of malaria in pregnancy using sulfadoxine-pyrimethamine in the Volta Region of Ghana
Source: PLoS One. 2024 Sep 5;19(9):e0308321. doi: 10.1371/journal.pone.0308321 (PMC11376531; doi:10.1371/journal.pone.0308321)
Supplement: Attachment — Submitted filename: Response_to_Reviewer_Report_LA.docx [file pone.0308321.s002.docx]

**RESPONSE TO REVIEWERS**

# Topic: **The demand for intermittent preventive treatment of malaria in pregnancy using sulfadoxine-pyrimethamine in the Volta Region of Ghana**

***Journal Requirements:***

1. When submitting your revision, we need you to address these additional requirements.

Please ensure that your manuscript meets PLOS ONE's style requirements, including those for file naming. The PLOS ONE style templates can be found at 
https://journals.plos.org/plosone/s/file?id=wjVg/PLOSOne_formatting_sample_main_body.pdf and 
<https://journals.plos.org/plosone/s/file?id=ba62/PLOSOne_formatting_sample_title_authors_affiliations.pdf>

***Response: Style requirement revised, including file naming, manuscript_LA, Reviewers_response, etc.***

2. We note that your Data Availability Statement is currently as follows: "All relevant data are within the manuscript and its Supporting Information files."

Please confirm at this time whether or not your submission contains all raw data required to replicate the results of your study. Authors must share the “minimal data set” for their submission. PLOS defines the minimal data set to consist of the data required to replicate all study findings reported in the article, as well as related metadata and methods (https://journals.plos.org/plosone/s/data-availability#loc-minimal-data-set-definition).

For example, authors should submit the following data:

- The values behind the means, standard deviations and other measures reported;
- The values used to build graphs;
- The points extracted from images for analysis.

Authors do not need to submit their entire data set if only a portion of the data was used in the reported study.

If your submission does not contain these data, please either upload them as Supporting Information files or deposit them to a stable, public repository and provide us with the relevant URLs, DOIs, or accession numbers. For a list of recommended repositories, please see https://journals.plos.org/plosone/s/recommended-repositories.

If there are ethical or legal restrictions on sharing a de-identified data set, please explain them in detail (e.g., data contain potentially sensitive information, data are owned by a third-party organization, etc.) and who has imposed them (e.g., an ethics committee). Please also provide contact information for a data access committee, ethics committee, or other institutional body to which data requests may be sent. If data are owned by a third party, please indicate how others may request data access.

**Response: It now reads: “All relevant data are within the manuscript”**

3. Please include captions for your Supporting Information files at the end of your manuscript, and update any in-text citations to match accordingly. Please see our Supporting Information guidelines for more information: <http://journals.plos.org/plosone/s/supporting-information>.

***Response: There is no supporting information apart those specify to be submitted***

4. Please review your reference list to ensure that it is complete and correct. If you have cited papers that have been retracted, please include the rationale for doing so in the manuscript text, or remove these references and replace them with relevant current references. Any changes to the reference list should be mentioned in the rebuttal letter that accompanies your revised manuscript. If you need to cite a retracted article, indicate the article’s retracted status in the References list and also include a citation and full reference for the retraction notice.

Additional Editor Comments:

Please address the comments by the reviewers

***Response: All reference materials cited in the work for [1] to [40]***

**Reviewer #1:**

Was there any social and behaviour change communication in the study area? If the answer is yes, was attendance at these activities one of the factors that could have affected women's attendance rate at ANC clinics?
Minor edits:
53 : survey, of = remove the comma

***Response: Line 51: Comma removed from line 57***

59: the results are presented

***Response: Line 63: the results were presented in the form of tables***

102: management, prevention =management and prevention

***Response: line 111 changed to: management and prevention***

128: malaria organisms = malaria parasites

***Response: line 140 changed to malaria parasites***

136: (2015). In addressing…

***Response: Line 148: change effected***

158: both districts were selected

***Response: Line 175: both districts were selected***

263: Ethical clearance was obtained

***Response: Line 280: Ethical clearance was obtained***

278: respondents

***Response: Line 299: respondents***

**Reviewer #2**

Your manuscript is well-established, but it needs some improvements before a final decision can be made. I have listed my comments, questions, and suggestions below:

1- The background section of the Abstract needs to be improved and redesigned to be clearer and more informative.

***Response: there is some improvement in the background with line 54-56 now read “This study sought to examine the uptake and service user predictors of the implementation of IPTp-SP after the policy upgrade in 2014”***

2- What is the rationale for choosing these two regions for this study?

***Response: Line 174-180: the rationale for choosing the two districts was based on their coverage and presents a peculiar and widespread case of the problem under investigation.***

3- In several parts of the manuscript, you used data up to 2017. Is there any updated data available for these sections?

***Response: Line 192: The data was collected data collect was done at 2020 at that time these were the references available, however, a few updates were done.***

4- Please recheck all the scientific names throughout the manuscript.

***Response: Line 143: all the scientific names throughout the manuscripts were checked and the correct ones used***

5- Include specific criteria for inclusion and exclusion in the study design.

***Response: Line 194-199 : the criteria for inclusion and exclusion now included in the study***

6- Add a citation for Yamane (1967).

***Response: citation for Yamane (1967) now done***

7- Use "STATA" instead of "Stata."

**Response: Line 252 change effected as STATA**

8- Provide information on the supplier at L221.

***Response: action taken***

9- Use only numeric values for percentages throughout the manuscript.

***Response: Line 327 change effected***

10- What do JHS and JSS stand for?

***Response: Line 313: change effected as “Junior High School/Junior Secondary School”***

11- Revise the section "Sociodemographic characteristics of the service users" for better clarity.

***Response: it is better with some revisions***

12- Add a well-known currency alongside the national currency for better impact.

***Response: Line 321: Dollar ($) was used***

13- What does "RAs" refer to at L400?

***Response: Line 453: Research Assistants (RA)***

**Reviewer #3:**

The analyzed information in this manuscript is valuable for CDC of Ghana, and helps to improve the management of malaria control during pregnancy in this country. I think this manuscript is suitable for publication in a regional Journal of Ghana.

***Response: Well, noted, however good journal of this nature will be useful.***

**Reviewer #4:**

I appreciate the opportunity to review the manuscript with the title “The demand for intermittent preventive treatment of 5 malaria in pregnancy using sulfadoxine-pyrimethamine in 6 the Volta Region of Ghana.” I commend the authors for a number of strengths of their work, including:
1. Managing to conduct this study given that, they did not have specific funding support to implement it.
2. The study aims of assessing predictors of IPTp-SP uptake which is one of the major challenges facing most malaria endemic countries towards meeting the set nationals and global targets. Observed issues will help informing the policy towards reviewing the existing strategies
3. Managed to articulate key predictors to determine their relationship with outcome variable
However, given the strengths which I have indicated, as I read the manuscript, I noted some areas in which I would have appreciated more clarification. I hope the paper could be further improved on:

1. Abstract:

Comments: Line number 53 can be rephrased to “*This cross-sectional survey was carried out in two selected districts in the Volta Region.”*

***Response: Line 57: Change effected***

Comments: Line number *58:* What has been done in your work is multiple logistic regression because it involves single outcome and several explanatory variables. Therefore, I recommend to mention “*Multiple* logistic regression analysis was used to determine …

***Response: Line 61-62 change effected***

2. Background section:

Comments: Line 75 - 93, provide details for malaria in general, which can distract a reader who is interested in malaria in pregnancy. Therefore, I would recommend to focus on the scope of the paper

Re***sponse: Part removed to refocus on the subject matter***

Comments: Line 117 – 118, the provided figure does not have reference to support it. Maybe it was an oversight.

***Response: It is now available***

Comments: Line 121 – 122, key references are missing

***Response: change effected***

Comments: Line 126 – 127, authors indicated that IPTp-SP was introduced because it is “*grounded in the belief that each pregnant woman in a malaria-endemic zone has malaria organisms in her blood and/or placenta.”* This does not seem to be the case. A lot of evidences have been generated indicating malaria parasites tending to harbor in the placenta. So, providing SP helps to reduce unwanted outcomes among others severe maternal anemia, miscarriages, underweight etc.

***Response: Line 156 change effected***

3. Methods section:

Comments: Line 154 – 158, and 162 – 166: These lines does not seem to be part of the methodology section; rather I would recommend to move them to the background. This is why background was overcrowded with lots of details which are not specific to the study topic while missing this crucial information.

***Response: Yes, I have checked and made the needed correction***

Comments: Line 192: I understand the provided formula was used to determine studied informants. However, I was wondering how this formula factored in the issue of the current status of IPTp uptake in the study area.

Where is the source of the indicated level of precision?

***Response: Yes, this formula factored issues of current status by adding 10% non-response to the estimated sample size at a precision of 5%.***

Comments: Line Line 195: The indicated sample frame (target) population which you indicated was estimated to be 76,341; kindly specify whether this is for entire Volta region or just in the studied districts.

In this section, I could not find how the surveyed districts were selected though, I noted in the conclusions that the districts were purposively selected

***Response: Line 243: This is for the entire Volta Region***

4. Results section:

Comments: Line Line 275: JHS/JSS this abbreviation appeared for the first time and I could not find anywhere written in its long form

***Response: Change effect in line 329***

Comments: Line 310: You indicated maternal age to have no effect on the uptake. However, in Line 314 – 316; you mentioned that “the higher the age the more the uptake. So, please could you please cross-check this to avoid confusing the reader.

***Response: Line 383: removed***

Comments: Line 340, 341: write in complete form in the bracket e.g., instead of *Crude odds ratio (OR)*; write Crude odds ratio (COR) and adjust as well

***Response: Line 412-417 change effect on Table 3***

5. Discussion section:

Comments: Line 364 – 367: In this section, you indicated pregnant women booking with gestational age above 16 weeks; their odds of getting 3+ doses were low. Again, this is contrary to what you indicated in Line 311 which you mentioned gestational age at booking for ANC to have no effect on the uptake. Kindly reconfirm your bivariate outputs.

***Response: Line 383 removed***

6. Limitation section:

Comments: Line 400: Ras in mentioned for the first time with no long form

***Response: Line 487: Research Assistants (RAs)***
