# Supplementary material for: The demand for intermittent preventive treatment of malaria in pregnancy using sulfadoxine-pyrimethamine in the Volta Region of Ghana
Source: PLoS One. 2024 Sep 5;19(9):e0308321. doi: 10.1371/journal.pone.0308321 (PMC11376531; doi:10.1371/journal.pone.0308321)
Supplement: Peer review history

# Peer review: Decision Letter 0

**DOI:** 10.1371/journal.pone.0308321.r001

**Authors:** Edison Arwanire Mworozi

15 Jul 2024

PONE-D-24-20857The demand for intermittent preventive treatment of malaria in pregnancy using sulfadoxine-pyrimethamine in the Volta Region of GhanaPLOS ONE

Dear Dr. Asem,

Thank you for submitting your manuscript to PLOS ONE. After careful consideration, we feel that it has merit but does not fully meet PLOS ONE’s publication criteria as it currently stands. Therefore, we invite you to submit a revised version of the manuscript that addresses the points raised during the review process.

Please address the comments from the reviewers accordingly.

Please submit your revised manuscript by Aug 29 2024 11:59PM. If you will need more time than this to complete your revisions, please reply to this message or contact the journal office at plosone@plos.org. When you're ready to submit your revision, log on to https://www.editorialmanager.com/pone/ and select the 'Submissions Needing Revision' folder to locate your manuscript file.

Please include the following items when submitting your revised manuscript:

- A rebuttal letter that responds to each point raised by the academic editor and reviewer(s). You should upload this letter as a separate file labeled 'Response to Reviewers'.
- A marked-up copy of your manuscript that highlights changes made to the original version. You should upload this as a separate file labeled 'Revised Manuscript with Track Changes'.
- An unmarked version of your revised paper without tracked changes. You should upload this as a separate file labeled 'Manuscript'.

If you would like to make changes to your financial disclosure, please include your updated statement in your cover letter. Guidelines for resubmitting your figure files are available below the reviewer comments at the end of this letter.

If applicable, we recommend that you deposit your laboratory protocols in protocols.io to enhance the reproducibility of your results. Protocols.io assigns your protocol its own identifier (DOI) so that it can be cited independently in the future. For instructions see: https://journals.plos.org/plosone/s/submission-guidelines#loc-laboratory-protocols. Additionally, PLOS ONE offers an option for publishing peer-reviewed Lab Protocol articles, which describe protocols hosted on protocols.io. Read more information on sharing protocols at https://plos.org/protocols?utm_medium=editorial-email&utm_source=authorletters&utm_campaign=protocols.

We look forward to receiving your revised manuscript.

Kind regards,

Edison Arwanire Mworozi, M.D

Academic Editor

PLOS ONE

Journal Requirements:

1. When submitting your revision, we need you to address these additional requirements.

Please ensure that your manuscript meets PLOS ONE's style requirements, including those for file naming. The PLOS ONE style templates can be found at 

https://journals.plos.org/plosone/s/file?id=wjVg/PLOSOne_formatting_sample_main_body.pdf and 

https://journals.plos.org/plosone/s/file?id=ba62/PLOSOne_formatting_sample_title_authors_affiliations.pdf

2. We note that your Data Availability Statement is currently as follows: "All relevant data are within the manuscript and its Supporting Information files."

Please confirm at this time whether or not your submission contains all raw data required to replicate the results of your study. Authors must share the “minimal data set” for their submission. PLOS defines the minimal data set to consist of the data required to replicate all study findings reported in the article, as well as related metadata and methods (https://journals.plos.org/plosone/s/data-availability#loc-minimal-data-set-definition).

For example, authors should submit the following data:

- The values behind the means, standard deviations and other measures reported;

- The values used to build graphs;

- The points extracted from images for analysis.

Authors do not need to submit their entire data set if only a portion of the data was used in the reported study.

If your submission does not contain these data, please either upload them as Supporting Information files or deposit them to a stable, public repository and provide us with the relevant URLs, DOIs, or accession numbers. For a list of recommended repositories, please see https://journals.plos.org/plosone/s/recommended-repositories.

If there are ethical or legal restrictions on sharing a de-identified data set, please explain them in detail (e.g., data contain potentially sensitive information, data are owned by a third-party organization, etc.) and who has imposed them (e.g., an ethics committee). Please also provide contact information for a data access committee, ethics committee, or other institutional body to which data requests may be sent. If data are owned by a third party, please indicate how others may request data access.

3. Please include captions for your Supporting Information files at the end of your manuscript, and update any in-text citations to match accordingly. Please see our Supporting Information guidelines for more information: http://journals.plos.org/plosone/s/supporting-information.

4. Please review your reference list to ensure that it is complete and correct. If you have cited papers that have been retracted, please include the rationale for doing so in the manuscript text, or remove these references and replace them with relevant current references. Any changes to the reference list should be mentioned in the rebuttal letter that accompanies your revised manuscript. If you need to cite a retracted article, indicate the article’s retracted status in the References list and also include a citation and full reference for the retraction notice.

Additional Editor Comments:

Please address the comments by the reviewers.

[Note: HTML markup is below. Please do not edit.]

Reviewers' comments:

Reviewer's Responses to Questions

**Comments to the Author**

1. Is the manuscript technically sound, and do the data support the conclusions?

The manuscript must describe a technically sound piece of scientific research with data that supports the conclusions. Experiments must have been conducted rigorously, with appropriate controls, replication, and sample sizes. The conclusions must be drawn appropriately based on the data presented. 

Reviewer #1: Yes

Reviewer #2: Partly

Reviewer #3: Partly

Reviewer #4: Yes

**********

2. Has the statistical analysis been performed appropriately and rigorously? 

Reviewer #1: Yes

Reviewer #2: Yes

Reviewer #3: I Don't Know

Reviewer #4: I Don't Know

**********

3. Have the authors made all data underlying the findings in their manuscript fully available?

The PLOS Data policy requires authors to make all data underlying the findings described in their manuscript fully available without restriction, with rare exception (please refer to the Data Availability Statement in the manuscript PDF file). The data should be provided as part of the manuscript or its supporting information, or deposited to a public repository. For example, in addition to summary statistics, the data points behind means, medians and variance measures should be available. If there are restrictions on publicly sharing data—e.g. participant privacy or use of data from a third party—those must be specified.

Reviewer #1: Yes

Reviewer #2: Yes

Reviewer #3: Yes

Reviewer #4: Yes

**********

4. Is the manuscript presented in an intelligible fashion and written in standard English?

PLOS ONE does not copyedit accepted manuscripts, so the language in submitted articles must be clear, correct, and unambiguous. Any typographical or grammatical errors should be corrected at revision, so please note any specific errors here.

Reviewer #1: Yes

Reviewer #2: Yes

Reviewer #3: Yes

Reviewer #4: Yes

**********

5. Review Comments to the Author

Please use the space provided to explain your answers to the questions above. You may also include additional comments for the author, including concerns about dual publication, research ethics, or publication ethics. (Please upload your review as an attachment if it exceeds 20,000 characters)

Reviewer #1: Was there any social and behaviour change communication in the study area? If the answer is yes, was attendance at these activities one of the factors that could have affected women's attendance rate at ANC clinics?

Minor edits:

53 : survey, of = remove the comma

59: the results are presented

102: management, prevention =management and prevention

128: malaria organisms = malaria parasites

136: (2015). In addressing…

158: both districts were selected

263: Ethical clearance was obtained

278: respondents

Reviewer #2: Dear Authors,

Your manuscript is well-established, but it needs some improvements before a final decision can be made. I have listed my comments, questions, and suggestions below:

1- The background section of the Abstract needs to be improved and redesigned to be clearer and more informative.

2- What is the rationale for choosing these two regions for this study?

3- In several parts of the manuscript, you used data up to 2017. Is there any updated data available for these sections?

4- Please recheck all the scientific names throughout the manuscript.

5- Include specific criteria for inclusion and exclusion in the study design.

6- Add a citation for Yamane (1967).

7- Use "STATA" instead of "Stata."

8- Provide information on the supplier at L221.

9- Use only numeric values for percentages throughout the manuscript.

10- What do JHS and JSS stand for?

11- Revise the section "Sociodemographic characteristics of the service users" for better clarity.

12- Add a well-known currency alongside the national currency for better impact.

13- What does "RAs" refer to at L400?

Reviewer #3: The analyzed information in this manuscript is valuable for CDC of Ghana, and helps to improve the management of malaria control during pregnancy in this country. I think this manuscript is suitable for publication in a regional Journal of Ghana.

Reviewer #4: I appreciate the opportunity to review the manuscript with the title “The demand for intermittent preventive treatment of 5 malaria in pregnancy using sulfadoxine-pyrimethamine in 6 the Volta Region of Ghana.” I commend the authors for a number of strengths of their work, including:

1. Managing to conduct this study given that, they did not have specific funding support to implement it.

2. The study aims of assessing predictors of IPTp-SP uptake which is one of the major challenges facing most malaria endemic countries towards meeting the set nationals and global targets. Observed issues will help informing the policy towards reviewing the existing strategies

3. Managed to articulate key predictors to determine their relationship with outcome variable

However, given the strengths which I have indicated, as I read the manuscript, I noted some areas in which I would have appreciated more clarification. I hope the paper could be further improved on:

1. Abstract:

Line number 53 can be rephrased to “This cross-sectional survey was carried out in two selected districts in the Volta Region.”

Line number 58: What has been done in your work is multiple logistic regression because it involves single outcome and several explanatory variables. Therefore, I recommend to mention “Multiple logistic regression analysis was used to determine …

2. Background section:

After going through the background section, I realized that a lot of information have been provided of which some are too general and not specific for the manuscript aim.

Line 75 - 93, provide details for malaria in general, which can distract a reader who is interested in malaria in pregnancy. Therefore, I would recommend to focus on the scope of the paper

Line 117 – 118, the provided figure does not have reference to support it. Maybe it was an oversight.

Line 121 – 122, key references are missing

Line 126 – 127, authors indicated that IPTp-SP was introduced because it is “grounded in the belief that each pregnant woman in a malaria-endemic zone has malaria organisms in her blood and/or placenta.” This does not seem to be the case. A lot of evidences have been generated indicating malaria parasites tend to harbor in the placenta. So, providing SP helps to reduce unwanted outcomes among others severe maternal anemia, miscarriages, underweight etc.

3. Methods section:

Line 154 – 158, and 162 – 166: These lines does not seem to be part of the methodology section; rather I would recommend to move them to the background. This is why background was overcrowded with lots of details which are not specific to the study topic while missing this crucial information.

Line 192: I understand the provided formula was used to determine studied informants. However, I was wondering how this formula factored in the issue of the current status of IPTp uptake in the study area.

Where is the source of the indicated level of precision?

Line 195: The indicated sample frame (target) population which you indicated was estimated to be 76,341; kindly specify whether this is for entire Volta region or just in the studied districts.

In this section, I could not find how the surveyed districts were selected though, I noted in the conclusions that the districts were purposively selected

4. Results section:

Line 275: JHS/JSS this abbreviation appeared for the first time and I could not find anywhere written in its long form

Line 310: You indicated maternal age to have no effect on the uptake. However, in Line 314 – 316; you mentioned that “the higher the age the more the uptake. So, please could you please cross-check this to avoid confusing the reader.

Line 340, 341: write in complete form in the bracket e.g., instead of Crude odds ratio (OR); write Crude odds ratio (COR) and for adjusted as well

5. Discussion section:

Line 364 – 367: This section you indicated pregnant women booking with gestational age above 16 weeks; their odds of getting 3+ doses were low. Again, this is contrary to what you indicated in Line 311 which you mentioned gestational age at booking for ANC to have no effect on the uptake. Kindly reconfirm your bivariate outputs.

6. Limitation section:

Line 400: Ras in mentioned for the first time with no long form

**********

6. PLOS authors have the option to publish the peer review history of their article (what does this mean?). If published, this will include your full peer review and any attached files.

If you choose “no”, your identity will remain anonymous but your review may still be made public.

**Do you want your identity to be public for this peer review?** For information about this choice, including consent withdrawal, please see our Privacy Policy.

Reviewer #1: **Yes: **Ahmed Adeel

Reviewer #2: No

Reviewer #3: No

Reviewer #4: **Yes: **Frank Chacky

**********

[NOTE: If reviewer comments were submitted as an attachment file, they will be attached to this email and accessible via the submission site. Please log into your account, locate the manuscript record, and check for the action link "View Attachments". If this link does not appear, there are no attachment files.]

While revising your submission, please upload your figure files to the Preflight Analysis and Conversion Engine (PACE) digital diagnostic tool, https://pacev2.apexcovantage.com/. PACE helps ensure that figures meet PLOS requirements. To use PACE, you must first register as a user. Registration is free. Then, login and navigate to the UPLOAD tab, where you will find detailed instructions on how to use the tool. If you encounter any issues or have any questions when using PACE, please email PLOS at figures@plos.org. Please note that Supporting Information files do not need this step.

Attachment Submitted filename: Review on the study conducted in Ghana _IPTp-SP.docx

# Author comment: Author response to Decision Letter 0

**DOI:** 10.1371/journal.pone.0308321.r002

17 Jul 2024

RESPONSE TO REVIEWERS

Topic: The demand for intermittent preventive treatment of malaria in pregnancy using sulfadoxine-pyrimethamine in the Volta Region of Ghana

Journal Requirements:

1. When submitting your revision, we need you to address these additional requirements.

Please ensure that your manuscript meets PLOS ONE's style requirements, including those for file naming. The PLOS ONE style templates can be found at 

https://journals.plos.org/plosone/s/file?id=wjVg/PLOSOne_formatting_sample_main_body.pdf and 

https://journals.plos.org/plosone/s/file?id=ba62/PLOSOne_formatting_sample_title_authors_affiliations.pdf

Response: Style requirement revised, including file naming, manuscript_LA, Reviewers_response, etc.

2. We note that your Data Availability Statement is currently as follows: "All relevant data are within the manuscript and its Supporting Information files."

Please confirm at this time whether or not your submission contains all raw data required to replicate the results of your study. Authors must share the “minimal data set” for their submission. PLOS defines the minimal data set to consist of the data required to replicate all study findings reported in the article, as well as related metadata and methods (https://journals.plos.org/plosone/s/data-availability#loc-minimal-data-set-definition).

For example, authors should submit the following data:

- The values behind the means, standard deviations and other measures reported;

- The values used to build graphs;

- The points extracted from images for analysis.

Authors do not need to submit their entire data set if only a portion of the data was used in the reported study.

If your submission does not contain these data, please either upload them as Supporting Information files or deposit them to a stable, public repository and provide us with the relevant URLs, DOIs, or accession numbers. For a list of recommended repositories, please see https://journals.plos.org/plosone/s/recommended-repositories.

If there are ethical or legal restrictions on sharing a de-identified data set, please explain them in detail (e.g., data contain potentially sensitive information, data are owned by a third-party organization, etc.) and who has imposed them (e.g., an ethics committee). Please also provide contact information for a data access committee, ethics committee, or other institutional body to which data requests may be sent. If data are owned by a third party, please indicate how others may request data access.

Response: It now reads: “All relevant data are within the manuscript”

3. Please include captions for your Supporting Information files at the end of your manuscript, and update any in-text citations to match accordingly. Please see our Supporting Information guidelines for more information: http://journals.plos.org/plosone/s/supporting-information.

Response: There is no supporting information apart those specify to be submitted

4. Please review your reference list to ensure that it is complete and correct. If you have cited papers that have been retracted, please include the rationale for doing so in the manuscript text, or remove these references and replace them with relevant current references. Any changes to the reference list should be mentioned in the rebuttal letter that accompanies your revised manuscript. If you need to cite a retracted article, indicate the article’s retracted status in the References list and also include a citation and full reference for the retraction notice.

Additional Editor Comments:

Please address the comments by the reviewers

Response: All reference materials cited in the work for [1] to [40]

Reviewer #1:

Was there any social and behaviour change communication in the study area? If the answer is yes, was attendance at these activities one of the factors that could have affected women's attendance rate at ANC clinics?

Minor edits:

53 : survey, of = remove the comma

Response: Line 51: Comma removed from line 57 

59: the results are presented

Response: Line 63: the results were presented in the form of tables

102: management, prevention =management and prevention

Response: line 111 changed to: management and prevention

128: malaria organisms = malaria parasites

Response: line 140 changed to malaria parasites

136: (2015). In addressing…

Response: Line 148: change effected

158: both districts were selected

Response: Line 175: both districts were selected

263: Ethical clearance was obtained

Response: Line 280: Ethical clearance was obtained

278: respondents

Response: Line 299: respondents

Reviewer #2

Your manuscript is well-established, but it needs some improvements before a final decision can be made. I have listed my comments, questions, and suggestions below:

1- The background section of the Abstract needs to be improved and redesigned to be clearer and more informative.

Response: there is some improvement in the background with line 54-56 now read “This study sought to examine the uptake and service user predictors of the implementation of IPTp-SP after the policy upgrade in 2014”

2- What is the rationale for choosing these two regions for this study?

Response: Line 174-180: the rationale for choosing the two districts was based on their coverage and presents a peculiar and widespread case of the problem under investigation.

3- In several parts of the manuscript, you used data up to 2017. Is there any updated data available for these sections?

Response: Line 192: The data was collected data collect was done at 2020 at that time these were the references available, however, a few updates were done.

4- Please recheck all the scientific names throughout the manuscript.

Response: Line 143: all the scientific names throughout the manuscripts were checked and the correct ones used

5- Include specific criteria for inclusion and exclusion in the study design.

Response: Line 194-199 : the criteria for inclusion and exclusion now included in the study

6- Add a citation for Yamane (1967).

Response: citation for Yamane (1967) now done

7- Use "STATA" instead of "Stata."

Response: Line 252 change effected as STATA

8- Provide information on the supplier at L221.

Response: action taken

9- Use only numeric values for percentages throughout the manuscript.

Response: Line 327 change effected

10- What do JHS and JSS stand for?

Response: Line 313: change effected as “Junior High School/Junior Secondary School”

11- Revise the section "Sociodemographic characteristics of the service users" for better clarity.

Response: it is better with some revisions 

12- Add a well-known currency alongside the national currency for better impact.

Response: Line 321: Dollar ($) was used

13- What does "RAs" refer to at L400?

Response: Line 453: Research Assistants (RA)

Reviewer #3: 

The analyzed information in this manuscript is valuable for CDC of Ghana, and helps to improve the management of malaria control during pregnancy in this country. I think this manuscript is suitable for publication in a regional Journal of Ghana.

Response: Well, noted, however good journal of this nature will be useful.

Reviewer #4: 

I appreciate the opportunity to review the manuscript with the title “The demand for intermittent preventive treatment of 5 malaria in pregnancy using sulfadoxine-pyrimethamine in 6 the Volta Region of Ghana.” I commend the authors for a number of strengths of their work, including:

1. Managing to conduct this study given that, they did not have specific funding support to implement it.

2. The study aims of assessing predictors of IPTp-SP uptake which is one of the major challenges facing most malaria endemic countries towards meeting the set nationals and global targets. Observed issues will help informing the policy towards reviewing the existing strategies

3. Managed to articulate key predictors to determine their relationship with outcome variable

However, given the strengths which I have indicated, as I read the manuscript, I noted some areas in which I would have appreciated more clarification. I hope the paper could be further improved on:

1. Abstract:

Comments: Line number 53 can be rephrased to “This cross-sectional survey was carried out in two selected districts in the Volta Region.”

Response: Line 57: Change effected

Comments: Line number 58: What has been done in your work is multiple logistic regression because it involves single outcome and several explanatory variables. Therefore, I recommend to mention “Multiple logistic regression analysis was used to determine …

Response: Line 61-62 change effected

2. Background section:

Comments: Line 75 - 93, provide details for malaria in general, which can distract a reader who is interested in malaria in pregnancy. Therefore, I would recommend to focus on the scope of the paper

Response: Part removed to refocus on the subject matter

Comments: Line 117 – 118, the provided figure does not have reference to support it. Maybe it was an oversight.

Response: It is now available

Comments: Line 121 – 122, key references are missing

Response: change effected

Comments: Line 126 – 127, authors indicated that IPTp-SP was introduced because it is “grounded in the belief that each pregnant woman in a malaria-endemic zone has malaria organisms in her blood and/or placenta.” This does not seem to be the case. A lot of evidences have been generated indicating malaria parasites tending to harbor in the placenta. So, providing SP helps to reduce unwanted outcomes among others severe maternal anemia, miscarriages, underweight etc.

Response: Line 156 change effected

3. Methods section:

Comments: Line 154 – 158, and 162 – 166: These lines does not seem to be part of the methodology section; rather I would recommend to move them to the background. This is why background was overcrowded with lots of details which are not specific to the study topic while missing this crucial information.

Response: Yes, I have checked and made the needed correction

Comments: Line 192: I understand the provided formula was used to determine studied informants. However, I was wondering how this formula factored in the issue of the current status of IPTp uptake in the study area.

Where is the source of the indicated level of precision?

Response: Yes, this formula factored issues of current status by adding 10% non-response to the estimated sample size at a precision of 5%. 

Comments: Line Line 195: The indicated sample frame (target) population which you indicated was estimated to be 76,341; kindly specify whether this is for entire Volta region or just in the studied districts.

In this section, I could not find how the surveyed districts were selected though, I noted in the conclusions that the districts were purposively selected

Response: Line 243: This is for the entire Volta Region

4. Results section:

Comments: Line Line 275: JHS/JSS this abbreviation appeared for the first time and I could not find anywhere written in its long form

Response: Change effect in line 329

Comments: Line 310: You indicated maternal age to have no effect on the uptake. However, in Line 314 – 316; you mentioned that “the higher the age the more the uptake. So, please could you please cross-check this to avoid confusing the reader.

Response: Line 383: removed

Comments: Line 340, 341: write in complete form in the bracket e.g., instead of Crude odds ratio (OR); write Crude odds ratio (COR) and adjust as well

Response: Line 412-417 change effect on Table 3

5. Discussion section:

Comments: Line 364 – 367: In this section, you indicated pregnant women booking with gestational age above 16 weeks; their odds of getting 3+ doses were low. Again, this is contrary to what you indicated in Line 311 which you mentioned gestational age at booking for ANC to have no effect on the uptake. Kindly reconfirm your bivariate outputs.

Response: Line 383 removed

6. Limitation section:

Comments: Line 400: Ras in mentioned for the first time with no long form

Response: Line 487: Research Assistants (RAs)

Attachment Submitted filename: Response_to_Reviewer_Report_LA.docx

# Editor report: Decision Letter 1

**DOI:** 10.1371/journal.pone.0308321.r003

**Authors:** Edison Arwanire Mworozi

23 Jul 2024

The demand for intermittent preventive treatment of malaria in pregnancy using sulfadoxine-pyrimethamine in the Volta Region of Ghana

PONE-D-24-20857R1

Dear Dr. Asem,

We’re pleased to inform you that your manuscript has been judged scientifically suitable for publication and will be formally accepted for publication once it meets all outstanding technical requirements.

Within one week, you’ll receive an e-mail detailing the required amendments. When these have been addressed, you’ll receive a formal acceptance letter and your manuscript will be scheduled for publication.

An invoice will be generated when your article is formally accepted. Please note, if your institution has a publishing partnership with PLOS and your article meets the relevant criteria, all or part of your publication costs will be covered. Please make sure your user information is up-to-date by logging into Editorial Manager at Editorial Manager® and clicking the ‘Update My Information' link at the top of the page. If you have any questions relating to publication charges, please contact our Author Billing department directly at authorbilling@plos.org.

If your institution or institutions have a press office, please notify them about your upcoming paper to help maximize its impact. If they’ll be preparing press materials, please inform our press team as soon as possible -- no later than 48 hours after receiving the formal acceptance. Your manuscript will remain under strict press embargo until 2 pm Eastern Time on the date of publication. For more information, please contact onepress@plos.org.

Kind regards,

Edison Arwanire Mworozi, M.D

Academic Editor

PLOS ONE

Additional Editor Comments (optional):

Reviewers' comments:

# Editor report: Acceptance letter

**DOI:** 10.1371/journal.pone.0308321.r004

**Authors:** Edison Arwanire Mworozi

25 Jul 2024

PONE-D-24-20857R1 

PLOS ONE

Dear Dr. Asem, 

I'm pleased to inform you that your manuscript has been deemed suitable for publication in PLOS ONE. Congratulations! Your manuscript is now being handed over to our production team.

At this stage, our production department will prepare your paper for publication. This includes ensuring the following:

* All references, tables, and figures are properly cited

* All relevant supporting information is included in the manuscript submission,

* There are no issues that prevent the paper from being properly typeset

If revisions are needed, the production department will contact you directly to resolve them. If no revisions are needed, you will receive an email when the publication date has been set. At this time, we do not offer pre-publication proofs to authors during production of the accepted work. Please keep in mind that we are working through a large volume of accepted articles, so please give us a few weeks to review your paper and let you know the next and final steps. 

Lastly, if your institution or institutions have a press office, please let them know about your upcoming paper now to help maximize its impact. If they'll be preparing press materials, please inform our press team within the next 48 hours. Your manuscript will remain under strict press embargo until 2 pm Eastern Time on the date of publication. For more information, please contact onepress@plos.org.

If we can help with anything else, please email us at customercare@plos.org.

Thank you for submitting your work to PLOS ONE and supporting open access. 

Kind regards, 

PLOS ONE Editorial Office Staff

on behalf of

Professor Edison Arwanire Mworozi 

Academic Editor

PLOS ONE
